# Trapeziectomy for basal thumb osteoarthritis does not increase the risk of developing wrist osteoarthritis in the long term

**DOI:** 10.1186/s13018-021-02856-x

**Published:** 2021-12-07

**Authors:** Elisabeth Brogren, Jack Besjakov, Anna Åkesson, Isam Atroshi

**Affiliations:** 1 Department of Translational Medicine, Lund University, 22100 Lund, Sweden; 2 Department of Hand Surgery, Skåne University Hospital, Malmö, Sweden; 3 Department of Diagnostic Radiology, Skåne University Hospital, Malmö, Sweden; 4 Clinical Studies Sweden-Forum South, Skåne University Hospital, Lund, Sweden; 5 Department of Clinical Sciences – Orthopedics, Lund University, Lund, Sweden; 6 Department of Orthopedics, Hässleholm-Kristianstad Hospitals, Hässleholm, Sweden

**Keywords:** Thumb basal joint osteoarthritis, Trapeziometacarpal joint osteoarthritis, Trapeziectomy, Long-term follow-up, Wrist osteoarthritis

## Abstract

**Background:**

Symptomatic osteoarthritis of the basal joint of the thumb (trapeziometacarpal joint) is a common disabling condition mainly affecting women. It is frequently treated with complete removal of the trapezium with or without soft-tissue interposition. There is limited evidence about whether removal of the trapezium affects stability of the wrist joint and increases the risk of developing wrist osteoarthritis. The aim of this study was to evaluate the long-term prevalence of OA in wrists with previous trapeziectomy compared to wrists with intact trapezium.

**Methods:**

Patients treated with surgery for trapeziometacarpal osteoarthritis at one orthopedic department were invited 10–29 (mean 17) years postoperatively for bilateral radiographic examination. We included radiographs from 114 hands with trapeziectomy and 46 hands with intact trapezium; 38 patients had unilateral trapeziectomy and intact contralateral trapezium. The radiographs were blinded so that the intact trapezium or the trapezial space after trapeziectomy was not visible. The radiographs were then evaluated for radiocarpal/midcarpal osteoarthritis independently by two assessors using three different osteoarthritis grading systems, including the Kellgren–Lawrence classification. The patients rated their satisfaction with the function of each of their hands on a visual analog scale (VAS) from 0 to 100 (higher score better).

**Results:**

The prevalence of osteoarthritis ranged from 20 to 26%, mostly mild (Kellgren–Lawrence grade 1). The prevalence of osteoarthritis did not differ between wrists with previous trapeziectomy and those with intact trapezium, both in the whole cohort and in the subgroup of patients with unilateral trapeziectomy and intact contralateral trapezium. There was no significant difference in hand function VAS scores between hands with previous trapeziectomy and hands with intact trapezium in the whole cohort or in the subgroup.

**Conclusions:**

Removal of the trapezium as treatment for basal thumb osteoarthritis does not increase the risk of developing wrist osteoarthritis in the long term.

## Background

Osteoarthritis (OA) of the thumb trapeziometacarpal (TMC) joint is a common disabling condition, mainly affecting women above age 50 [1]. Various surgical procedures are used to treat symptomatic TMC OA [2, 3], the most common involve trapeziectomy, either alone or combined with ligament reconstruction and tendon interposition (LRTI) or combined with tendon suspension-interposition [2, 3]. In the United States, trapeziectomy with LRTI is one of the most common procedures used for treating TMC OA [2]. Trapeziectomy partly disrupts the constrains of the scaphotrapeziotrapeziod (STT) joint, which has been suggested to potentially cause a carpal instability pattern that has been termed as non-dissociative [4–6]. Non-dissociative carpal instability is defined as a kinematic dysfunction of the entire proximal carpal row, manifested as either an instability between the proximal and distal rows, or between the proximal row and the radius [7]. A cadaveric study has demonstrated that the STT ligament is one of the important stabilizers of the proximal carpal row [8]. Previous reports of patients with dysfunction of the STT ligament, either secondary to severe STT OA or distal scaphoid excision, demonstrated development of non-dissociative carpal instability pattern with extension of the proximal carpal row in relation to the distal row [9, 10]. A recent study suggested that trapeziectomy in combination with partial or complete trapezoidectomy increased the risk of developing non-dissociative carpal instability and worse functional outcome seven years after surgery [11].

In dissociative carpal instability, there is a disruption of intrinsic intercarpal ligaments causing instability between bones within the same carpal row (i.e., scapholunate or lunotriquetral) [12]. It is well-known that scaphoid nonunion and dissociative instability caused by disruption of the SL ligament commonly result in secondary wrist OA in the long term [13]. However, it is not known whether potential distal destabilization of the scaphoid through disruption of the STT ligament and removal of the bony support provided by the trapezium may result in long-term wrist OA. The possible long-term adverse effect of trapeziectomy has been one of the arguments behind the continuous introduction of new TMC implants, often associated with very high costs and substantial complications [14, 15]. Whether potential carpal instability after trapeziectomy could lead to increased risk of developing OA in adjacent carpal joints can only be assessed with long-term follow-up.

The aim of this study was to compare the long-term prevalence of wrist OA in patients previously treated with trapeziectomy versus wrists with intact trapezium.

## Methods

In this single-center cohort study, we searched the surgery register for one university orthopedic department (Hässleholm and Kristianstad Hospitals) in southern Sweden in August 2018 and identified all adults who had been treated with any type of surgical procedure with the diagnosis code for TMC OA (ICD-10 code M18.0 and M18.1) from January 1, 1998 through December 31, 2005. The study center is the main health care facility to which patients with TMC OA are referred and the only center where surgery for TMC OA is performed in a region with 300,000 inhabitants. The primary purpose of the research, as described previously [16], was to study the long-term clinical and radiological outcomes after surgery for TMC OA. In the current study, we aimed to evaluate the prevalence of radiographic wrist OA 10 years or longer after trapeziectomy. The study was approved by the Ethical Review Board of Lund University, Lund, Sweden (#2018/485) and written informed consent was obtained from all participants.

The inclusion criteria for this study were hands that had undergone trapeziectomy at least 10 years before the radiographic examination (i.e., including those performed before or after the study period) or hands with intact trapezium (i.e., no previous surgical procedures involving the trapezium). The exclusion criteria were hands with trapeziectomy performed less than 10 years before examination (because possible development of OA usually occurs over several years), hands with TMC fusion, and total or partial wrist fusion. All hands that fulfilled the eligibility criteria were included in this study.

**Fig. 1:**
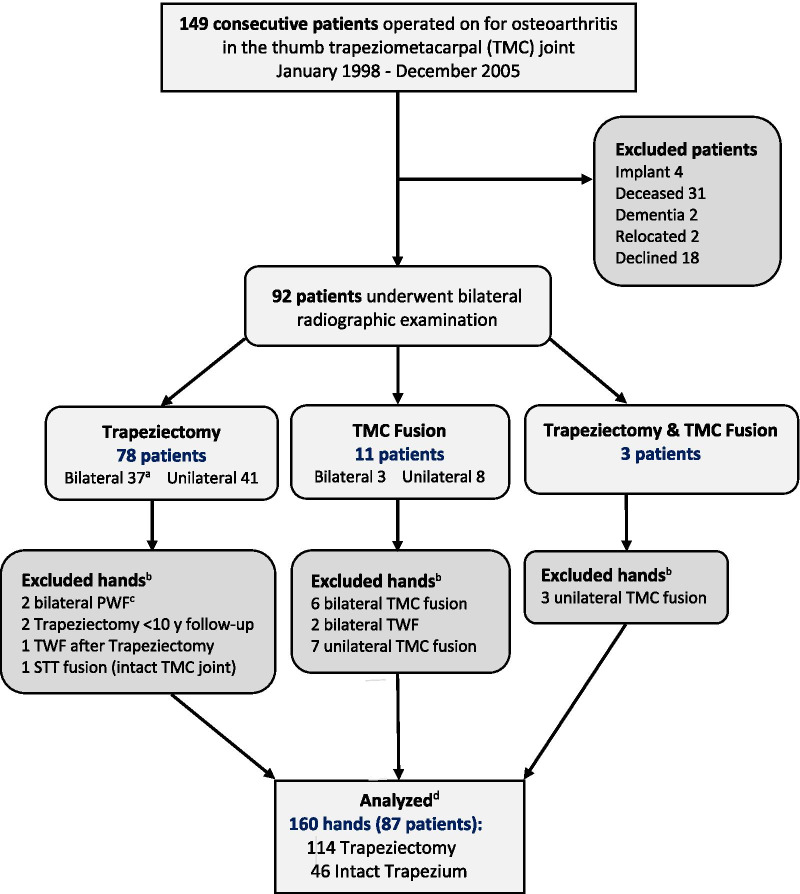
Study enrollment flowchart. ^a^16 patients operated on with unilateral trapeziectomy during 1998–2005 had contralateral trapeziectomy before 1998 or after 2005. ^b^Exclusion criteria: Trapeziectomy performed less than 10 years before examination, trapeziometacarpal (TMC) fusion, scaphotrapeziotrapezoidal (STT) fusion, partial wrist fusion (PWF) or total wrist fusion (TWF). ^c^Scapho-trapezoid-capitate fusion 4 years after unilateral trapeziectomy followed by same procedure contralaterally (radiographs at this follow-up show no radiocarpal OA in any side). ^d^Hands (operated and non-operated) that met the inclusion criteria (trapeziectomy or intact trapezium) and had none of the exclusion criteria

We identified 149 consecutive patients who had undergone surgery for TMC OA during the 8-year study period. Of these, 31 patients had died and 4 patients with implant arthroplasty were excluded. The remaining 114 patients were asked to attend the hospital for clinical and radiographic examination. Two patients had developed dementia and lived in a care facility and two patients had moved to unknown addresses and could not be contacted. Of the remaining 110 patients, 18 responded to mailed questionnaires or telephone interview but declined radiographic examination. Thus, 92 patients operated on with unilateral or bilateral trapeziectomy or TMC fusion during the study period attended bilateral radiographic hand/wrist examination performed September–December 2018. At the time of the examinations, 10 of the patients who had unilateral trapeziectomy during the study period had undergone contralateral trapeziectomy after 2005 (Fig. 1).

The final cohort comprised 87 patients (160 eligible hands), of whom 35 patients (70 hands) with bilateral trapeziectomy, 38 patients with unilateral trapeziectomy (38 hands) and intact contralateral trapezium (38 hands), 6 patients with trapeziectomy on one side (6 hands), and 8 patients with intact trapezium on one side (8 hands). Thus, the analysis includes 114 hands with trapeziectomy and 46 hands with intact trapezium (Fig. 1). None of the patients had been treated with simultaneous trapezoidectomy.

### Radiography

**Fig. 2:**
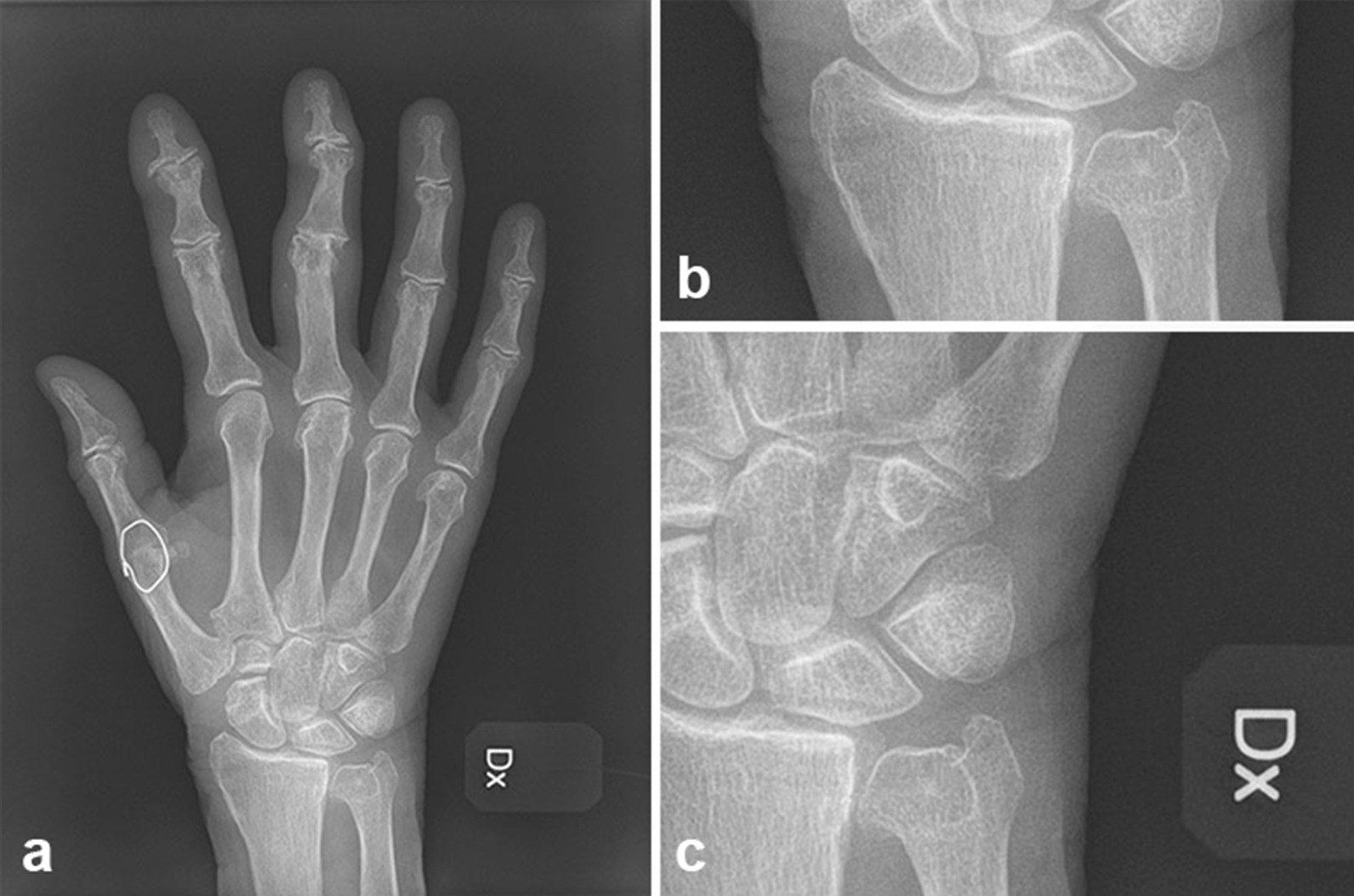
Radiograph of wrist 18 years after removal of the trapezium, before blinding (**a**), and after blinding (**b**, **c**)

**Fig. 3:**
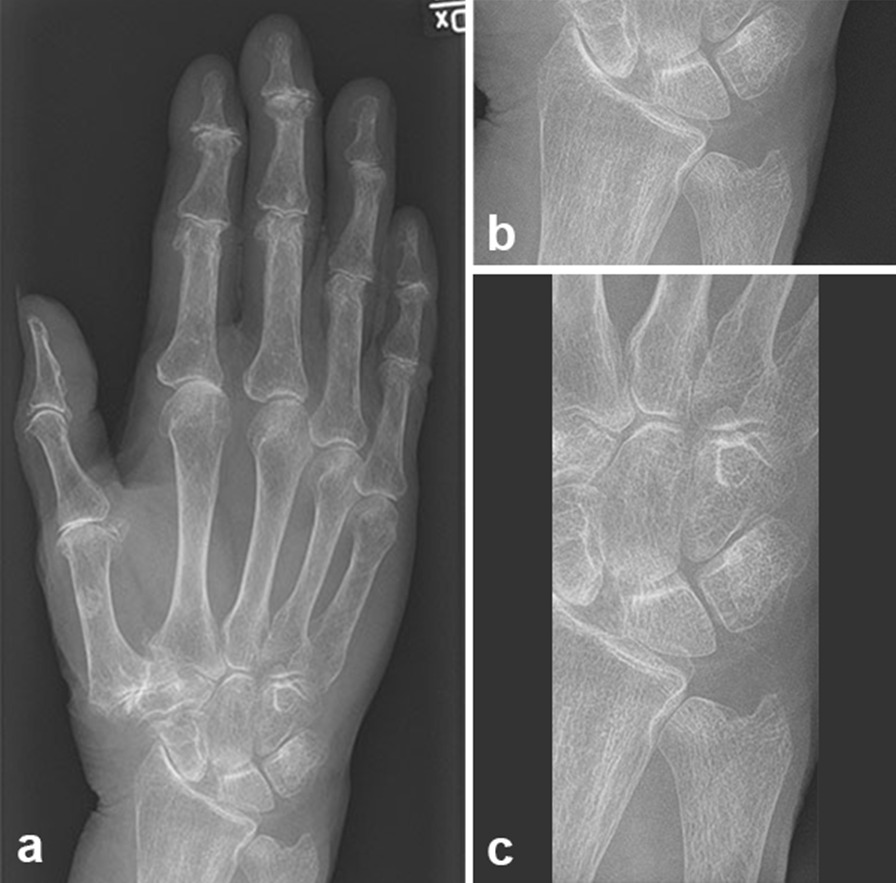
Radiograph of wrist with intact trapezium (ie, no previous surgery), before blinding (**a**), and after blinding (**b**, **c**)

Radiographic examination of both wrists was performed at the time of this follow-up. A research assistant anonymized and cropped the digital radiographs (posteroanterior views) to only show the radiocarpal and intercarpal joints so that it was not possible to see whether the trapezium was present or absent. Each radiograph was cropped both horizontally and longitudinally, allowing most of the carpus to be visualized without showing the trapezium (if present) or the trapezial space (if trapezium absent) (Figs. 2, 3).

The radiographs (2 radiographs for every right and left wrist) were randomly coded using Excel random number function. The images were then examined independently on one occasion during September 2020, by two blinded assessors, who are experienced specialists in musculoskeletal radiography (JB) and hand surgery (EB). The two assessors had no knowledge of the number of wrists with or without trapeziectomy or which radiographs of right and left wrists belonged to the same individual.

### Definition of osteoarthritis

A priori we chose to use three OA grading systems to assess the presence of wrist OA on the radiographs: Kellgren–Lawrence (grade 0–4) [17], scapholunate advanced collapse (SLAC) (grade 0–4) [18], and 5-zone (joint narrowing, presence of osteophytes and sclerotic/cystic changes) in the radial styloid-scaphoid joint, entire radioscaphoid joint, radiolunate joint, scaphocapitate joint and capitolunate joint [19]. In the 5-zone system, we merged the 5 zones into 3 zones: the radioscaphoid (zone 1 and 2), the radiolunate (zone 3) and the midcarpal (zone 4 and 5). The two examiners also recorded whether they judged the scapholunate interval as increased (> 3 mm) or within normal limit.

### Satisfaction with hand function

The patients were asked to rate their satisfaction with the function of each of their hands, on average during the past week, on a visual analog scale (VAS) ranging from 0 (dissatisfied) to 100 (very satisfied).

### Statistical analyses

Data are shown as means and SD or proportions as appropriate. Because few cases had advanced OA according to all three grading systems, we classified each wrist as OA or no OA. We used the Chi-square test to compare the presence of OA (according to each of the three grading systems) in the trapeziectomy wrists versus the intact-trapezium wrists. As a subgroup analysis, we compared the presence of OA in the 38 patients with a removed trapezium on one side and intact trapezium on the other side using McNemar test. All analyses were performed using the radiographic measurements from the two blinded assessors separately. We also performed a sensitivity analysis considering OA present if both assessors graded present versus wrists graded as no OA by both assessors. A linear mixed-model analysis was performed to compare hand function VAS scores in the trapeziectomy hands and the intact-trapezium hands, adjusting for age, sex and dominance of the hand (dominant or non-dominant). The mixed model was chosen because it accounts for multiple-level data (patients providing data for more than one hand) and performed with Stat SE 16.1 (StatCorp, College Station, TX). In the subgroup of 38 patients, the paired t-test was used to compare the VAS scores in the trapeziectomy hands with the contralateral intact-trapezium hands. A *p* value < 0.05 was used for statistical significance.

## Results

Radiographic examination was conducted on 75 women and 12 men, with mean age of 75 (SD 6.5) years (Table 1). All except 2 were right-hand dominant. The mean time from trapeziectomy was 17 (SD 3.3) years. Of the 38 patients (32 women) who had trapeziectomy on one side and intact trapezium on the other side; 2 were left-hand dominant, of whom 1 had trapeziectomy on the dominant side.

**Table 1:** Patient characteristics

	Trapeziectomy			Trapezium intact			Subgroup trapeziectomy and contralateral trapezium intact		
	All	Women	Men	All	Women	Men	All	Women	Men
No. of hands	114	106	8	46	35	11	38	32	6
Right	20	18	2	23	16	7	18	16	2
Left	24	20	4	23	19	4	20	16	4
Bilateral	70	68	2	0	0	0	0	0	0
Age at trapeziectomy^a^	58 (44–72)	58 (44–72)	63 (50–71)	NA	NA	NA	60 (50–72)	59 (51–72)	62 (50–71)
Age at examination	75 (59–91)	74 (59–91)	83 (63–87)	75 (62–91)	75 (62–91)	74 (62–87)	75 (63–91)	75 (67–91)	81 (63–87)
Time since trapeziectomy^a^	16 (10–29)	16 (10–29)	19 (13–22)	NA	NA	NA	17 (13–21)	17 (13–21)	17 (13–19)

*NA* not applicable

^a^For the trapeziectomy hands. Values are median (range) years

### Prevalence of wrist osteoarthritis

The prevalence of OA ranged from 20 to 26%, mostly mild (Kellgren–Lawrence grade 1 and SLAC grade 1). In the entire cohort as well as in the subgroup of 38 patients with unilateral trapeziectomy and intact contralateral trapezium, the proportion of hands with OA did not differ between trapeziectomy wrists and intact-trapezium wrists (Table 2). The most common site for OA was the radioscaphoid joint; the difference in OA prevalence between the intact-trapezium group and the trapeziectomy group as evaluated by Assessor 1 was 1.5% (95% CI − 13 to 17%) and by Assessor 2 was 1.6% (95% CI − 12 to 16%). The sensitivity analysis did not show any differences between the two groups. None of the comparisons (trapeziectomy vs intact trapezium or increased vs normal scapholunate interval) was statistically significant (all *p* values > 0.1).

**Table 2:** The presence of radiographic wrist osteoarthritis evaluated independently by two blinded assessors

Osteoarthritis (OA) grading system	Assessor 1		Assessor 2	
	Trapeziectomy	Trapezium intact	Trapeziectomy	Trapezium intact
	*n (%), n*	*n (%), n*	*n (%), n*	*n (%), n*
Kellgren–Lawrence				
OA	28 (25), *10*	12 (26), *11*	23 (20), *8*	10 (22), *7*
No OA	86 (75), *28*	34 (74), *27*	91 (80), *30*	36 (78), *31*
SLAC				
OA	28 (25), *10*	12 (26), *11*	23 (20), *9*	10 (22), *9*
No OA	86 (75), *28*	34 (74), *27*	91 (80), *29*	36 (78), *29*
Five-zones				
Zone 1–2 (radioscaphoid)				
OA	28 (25), *10*	11 (24), *10*	23 (20), *9*	10 (22), *9*
No OA	86 (75), *28*	35 (76), *28*	91 (80), *29*	36 (78), *29*
Zone 3 (radiolunate)				
OA	2 (2), *1*	3 (6), *1*	6 (5), *0*	4 (9), *2*
No OA	112 (98), *37*	43 (94), *37*	108 (95), *38*	42 (91), *36*
Zone 4–5 (midcarpal)				
OA	12 (10), *4*	5 (11), *3*	26 (23), *9*	10 (22), *8*
No OA	102 (90), *34*	41 (89), *35*	88 (77), *29*	36 (78), *30*
Scapholunate interval				
Increased	14 (12), *6*	7 (15), *5*	14 (12), *8*	9 (20), *7*
Normal	100 (88), *32*	39 (85), *33*	100 (88), *30*	37 (80), *31*

Values for all 160 hands are presented

Figures in italic are number of hands in the subgroup of 38 patients with trapeziectomy on one side and intact trapezium on the other side

### Hand function scores

The adjusted mean VAS score was 76 (95% CI 72–81) in hands with previous trapeziectomy and 73 (95% CI 67–80) in hands with intact trapezium; adjusted mean difference 3.1 (95% CI − 3.5–9.7; *p* = 0.36).

No significant difference in the mean VAS score was found in the subgroup of 38 patients; mean VAS score was 76 (SD 19) in the hands with previous trapeziectomy and 72 (SD 28) in the hands with intact trapezium, mean difference 3.6 (95% CI − 6.1–13, *p* = 0.46).

## Discussion

This long-term follow-up of patients treated with trapeziectomy and tendon suspension-interposition arthroplasty showed no increased risk of developing radiographic OA in adjacent carpal joints. Although concerns have been raised that trapeziectomy could destabilize the carpus, this is to our knowledge the first long-term study that addresses the prevalence of wrist OA after trapeziectomy as compared to wrists with intact trapezium.

As an important stabilizer of both the scaphoid and the proximal row of the carpus, the STT ligament acts as a restraint to the development of a non-dissociative carpal instability pattern [8]. During trapeziectomy, the majority of the STT ligament is divided and left unrepaired, which potentially could destabilize the proximal carpal row [8]. A study that analyzed radiographs of 61 patients at mean 3.2 (0.3–13) years after trapeziectomy/LRTI did not find signs of radiographic wrist destabilization based on measurements of carpal height, radiolunate angle, scapholunate angle and ulnar translation of the carpus [20]. In contrast, a study that reviewed radiographs from 22 patients before and at mean of 8.5 months after trapeziectomy/LRTI reported a significant increase in number of wrists with abnormal scapholunate angle from 32% before to 55% after surgery [6]. In a study of 122 wrists examined before and at a mean of 3.5 (1–13) years after trapeziectomy/LRTI, the authors suggested increased risk of developing non-dissociative carpal instability patterns after trapeziectomy [4]. In a previous report of two cases of carpal collapse after trapeziectomy and partial trapezoid resection, the authors suggested that carpal collapse may more likely occur after trapeziectomy with simultaneous partial trapezoidectomy than after trapeziectomy alone [5]. A recently published 7-year follow-up study of trapeziectomy and partial trapezoidectomy (21 hands), trapeziectomy and complete trapezoidectomy (22 hands) and trapeziectomy and no trapezoidectomy (36 hands) found that compared to no trapezoidectomy, both partial and complete trapeziodectomy led to greater incidence of non-dissociative carpal instability and inferior functional results [11]. However, none of these studies assessed wrist OA or compared trapeziectomy with wrists in which the trapezium was intact. We did not investigate possible presence of carpal instability but did not find any increased prevalence of wrist OA 10 years or longer after trapeziectomy in a large cohort, assessed by two experienced blinded examiners using three different OA grading systems. In addition, the finding was consistent even in the subgroup that had trapeziectomy on one side and intact trapezium on the other side.

We used three different OA grading systems to evaluate the presence of OA because they have different features. The Kellgren–Lawrence OA grading system, one of the most commonly used systems for OA in all joints, is based on the presence of osteophytes, which may not always be applicable to midcarpal OA, where joint narrowing and subchondral sclerosis could be the only signs [17]. The radiological pattern of SLAC wrist includes a predictable progression through four stages [18]. However, Miller et al. [19] found that less than half of the patients with wrist OA fell into the category of SLAC arthritis and suggested a different grading scheme, in which degenerative changes in the midcarpal joints, without osteophytes and without concomitant radiocarpal OA, is included in the grading. The advantage of using the 5-zone grading system is that isolated degenerative changes in the radiolunate and/or midcarpal joints can be evaluated in the absence of a traditional SLAC arthritis pattern.

We found wrist OA in 20–26% of the examined wrists, with degenerative changes most often located in the radioscaphoid joint. The prevalence of radiological wrist OA in the general population or among persons with hand osteoarthritis in the general population is not known. A previous study examined 100 consecutive wrist radiographs from patients who had sought healthcare for general hand problems at one orthopedic department and found radiological TMC OA in 65% and wrist OA in 29% [21].

The main strength of our study is that the assessors were blinded to whether the trapezium had been removed or was intact. Other strengths are the long follow-up time and high participation rate (76% of the patients still alive). This study has limitations. We lacked access to preoperative radiographs and thus could not assess whether radiocarpal or midcarpal OA was present before trapeziectomy. Although preoperative radiographs could have been useful, most of the initial radiographs performed 1998–2005 were non-digital and may not be directly comparable with the digital long-term follow-up radiographs. Besides, since there was no difference in the prevalence of wrist OA between wrists with trapeziectomy and those with intact trapezium, preoperative arthritis status is not a major issue. Another possible limitation is use of plain radiographs to assess the presence of OA. Although CT scans or MRI could have provided additional information it may not be practical or ethically justifiable to subject large number of individuals, many asymptomatic and did not actively seek healthcare, to such extensive examinations. Besides, assessment of wrist OA on plain radiographs has shown good reliability [22].

In conclusion, this study shows that trapeziectomy does not increase the risk of developing wrist OA in the long term. This finding is important when choosing type of surgical treatment in patients with base of the thumb OA and when informing patients about their long-term prognosis.

## Data Availability

The data in this study are available from the corresponding author upon request, if legally and ethically possible.

## Abbreviations

LRTI: Ligament reconstruction and tendon interposition
OA: Osteoarthritis
SLAC: Scapholunate advance collapse
STT: Scaphotrapeziotrapeziod
TMC: Trapeziometacarpal
TMC OA: Trapeziometacarpal osteoarthritis
